# Raw Data-Based Motion Compensation for High-Resolution Sliding Spotlight Synthetic Aperture Radar

**DOI:** 10.3390/s18030842

**Published:** 2018-03-12

**Authors:** Ning Li, Shilin Niu, Zhengwei Guo, Yabo Liu, Jiaqi Chen

**Affiliations:** 1 School of Computer and Information Engineering, Henan University, Kaifeng 475004, China; nsl1993@foxmail.com (S.N.); henugzw@foxmail.com (Z.G.); 2 Space Microwave Remote Sensing System Department, Institute of Electronics, Chinese Academy of Sciences, Beijing 100190, China; ybliu@mail.ie.ac.cn; 3 College of Computer and Information Engineering, Hohai University, Nanjing 210098, China; jiaqichen@hhu.edu.cn

**Keywords:** motion compensation (MOCO), sliding spotlight, synthetic aperture radar (SAR), weighted total least square (WTLS) method, Doppler rate (DR)

## Abstract

For accurate motion compensation (MOCO) in airborne synthetic aperture radar (SAR) imaging, a high-precision inertial navigation system (INS) is required. However, an INS is not always precise enough or is sometimes not even included in airborne SAR systems. In this paper, a new, raw, data-based range-invariant motion compensation approach, which can effectively extract the displacements in the line-of-sight (LOS) direction, is proposed for high-resolution sliding spotlight SAR mode. In this approach, the sub-aperture radial accelerations of the airborne platform are estimated via a well-developed weighted total least square (WTLS) method considering the time-varying beam direction. The effectiveness of the proposed approach is validated by two airborne sliding spotlight C band SAR raw datasets containing different types of terrain, with a high spatial resolution of about 0.15 m in azimuth.

## 1. Introduction

SLIDING spotlight synthetic aperture radar (SAR) is designed to provide a compromise between spotlight and stripmap modes, where the rotation center of the antenna beam is located beyond the beam footprints, hence increasing the observed azimuth scene extension at the expense of azimuth resolution [1,2]. However, despite its merits, sliding spotlight SAR also had some problems that need to be solved. The first problem is that the total Doppler bandwidth is generally greater than the pulse repetition frequency (PRF), especially for the spaceborne case, due to the azimuth beam steering in the sliding spotlight mode [1,2]. To solve this problem, several efficient algorithms have been developed for sliding spotlight SAR data processing [3,4,5,6,7,8] such as the extended chirp scaling (ECS) algorithm [3], baseband azimuth scaling algorithm [4] and the two-step processing algorithm as well as its improvements [5,6,7,8], most of which are based on the conventional stripmap imaging algorithms. The second problem is that the motion errors arising from trajectory deviations, especially for the high-resolution spotlight or sliding spotlight airborne case, severely degrade the image quality both in geometric and radiometric resolution [9,10]. Therefore, motion compensation (MOCO) for the sliding spotlight mode is necessary to obtain the best possible image focusing.

The two-step motion compensation (MOCO) strategy is a well-established technique, which has been widely used for airborne SAR data processing [9,10,11,12]. In this strategy, the first-order MOCO compensates the range-invariant component of the motion errors; the second-order MOCO compensates the range-varying component of the residual motion errors. Both can be achieved using approaches based on inertial navigation systems (INS) or SAR raw data. Generally, a combination of both approaches is desired for MOCO. However, in many cases, an INS is not included or not precise enough in the SAR system due to the limit of the airborne platform, such as light weight aircrafts and unmanned aerial vehicles (UAV). Under this condition, raw data-based first-order MOCO techniques need to be developed to replace the function of INS, which also motivates the development of the method proposed in this paper.

In past years, several classical raw data-based MOCO approaches have been developed for both the stripmap and spotlight modes [13,14,15,16,17,18,19,20,21], including Doppler rate estimation autofocus (DRA) [13], phase gradient autofocus (PGA) [14], sharpness optimization autofocus (SOA) [15,16], and so on. In recent years, based on the aforementioned approaches, some methods, which can extract the displacements in line-of-sight (LOS) direction, have been developed [17,18,19,20,21]. In [17,18], the motion errors were extracted by the double integral of the estimated radial acceleration in LOS direction. In [19,20], the PGA technique is modified to have the capability to correct the excessive range cell migration (RCM) by adding proper weighting and filtering operations. More recently, an innovative Mapdirft-PGA (MD-PGA) technique was proposed [21], which can correct the excessive RCM and phase errors precisely in a sub-aperture manner.

In this paper, an improved raw data-based first-order MOCO (IRDM) approach is proposed for sliding spotlight mode, which is modified from previous work intended for stripmap mode [18]. Inspired by the work presented in [18], the LOS displacements were also extracted by the estimated radial accelerations. However, due to the time-varying beam direction in sliding spotlight mode, the Doppler rates (DRs) in different sub-apertures will be also influenced. The proposed method can calculate the radial accelerations of the airborne platform considering the time-varying DRs caused by the beam steering, which is the main contribution of the proposed method. In addition, a weighted total least square (WTLS) method and an iterative strategy are used to improve the estimation accuracy of the radial accelerations. After range compression operation, a range-dependent PGA in a sub-patched manner is exploited for second-order MOCO to correct residual phase errors [22]. High-resolution airborne sliding spotlight real datasets are used to test the proposed approach.

The manuscript is organized as follows. In Section 2, some related works are briefly reviewed. In Section 3, the proposed IRDM approach is presented in detail. In Section 4, the experimental results and the analysis based on real data are given. Finally, the conclusion is drawn in Section 5.

## 2. Related Works

### 2.1. Properties of Sliding Spotlight Mode

**Figure 1:**
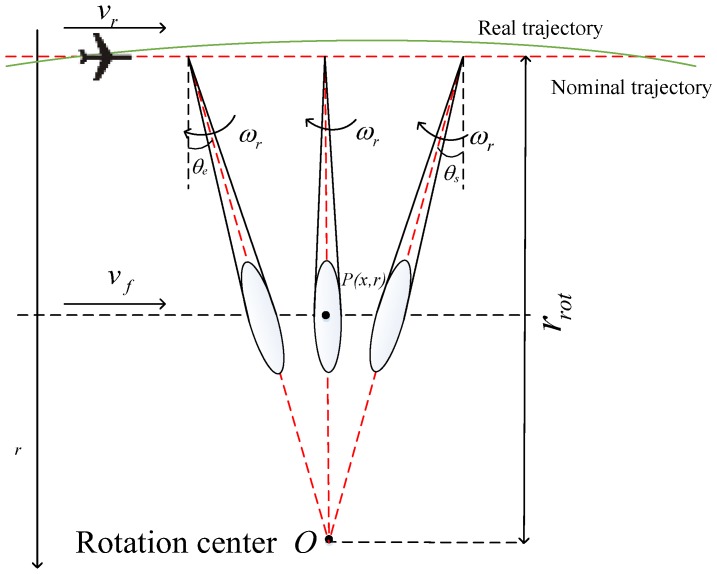
Planar imaging geometry of the airborne sliding spotlight mode.

The planar imaging geometry of the airborne sliding spotlight mode is shown in Figure 1, where the red straight line denotes the nominal trajectory, the green curve represents the real trajectory, the azimuth angle of the main beam is steered from $\theta_{s}$ to $\theta_{e}$ at a constant rotation rate $\omega_{r}$, the virtual rotation center O is positioned farther than the beam footprint, P is a point target in the imaged swath, r and $r_{rot}$ are the slant range from the flight path to the imaged target and the virtual rotation center, $v_{r}$ and $v_{f}$ are the velocity of the airborne platform and beam footprint, respectively. The beam steering factor A in the sliding spotlight can be expressed as [8]:

(1) $$A=\frac{v_{f}}{v_{r}}=\frac{r_{rot}-r}{r_{rot}}$$

The beam steering factor A is an important factor for the final obtained azimuth resolution in the sliding spotlight mode, the relationship between the sliding spotlight mode and the stripmap mode on the azimuth resolution can be approximately expressed as follows [8]:

(2) $$\rho_{a\_slide}=A\cdot \rho_{a\_strip}=\frac{r_{rot}-r}{r_{rot}}\cdot \frac{D}{2}$$

where D is the antenna length. From Equation (2), it can be seen that the obtained azimuth resolution changes with slant range in sliding spotlight mode, which is better in far range than in near range.

Compared to the signal format of the stripmap mode, the major difference in the sliding spotlight mode is its time-varying Doppler centroid caused by the beam steering, which results difficulties in data processing steps when directly using standard stripmap airborne processor. The varying rate of the Doppler centroid is given as follows:

(3) $$k_{rot}=\frac{\partial [f_{dc}(t)]}{\partial t}\approx \frac{2\cdot v_{r}^{2}}{\lambda \cdot r_{rot}}$$

where $f_{dc}\left(t\right)$ denotes the time-varying Doppler centroid.

### 2.2. RDM Approach for Stripmap Mode

In the RDM approach for the stripmap mode [18], a side-looking stripmap imaging geometry was assumed and the Doppler rate errors (DREs) were approximately expressed as:

(4) $$\Delta k_{a}(t_{m})\approx -\frac{2}{\lambda }a_{y}\sin\beta -\frac{2}{\lambda }a_{z}\cos\beta$$

where λ denotes the radar wavelength, β denotes the look angle, $a_{y}$ and $a_{z}$ denote the lateral and vertical accelerations in cross-track plane, respectively. Note that the range error introduced by the forward velocity was ignored here, due to the fact that the forward velocity of the airborne platform is generally with slow and small variations when radar data were collected. If the changes of forward velocity exceed the limitation, a re-sampling operation in azimuth can be done to eliminate the error.

Based on Equation (4), in the range compressed and azimuth-time domain, the data are segmented into overlapped sub-apertures in azimuth direction. The length of each sub-aperture should be much smaller than one synthetic aperture length (e.g., around one tenth of the total synthetic aperture length) so that the variation of DRs can be ignored. In each sub-aperture, contrast-optimized algorithm (COA) was used to estimate DRs of *N* different range bins. Then, the estimated DREs in nth range bin and mth azimuth sub-aperture can be expressed as follows:

(5) $$\Delta {\hat{k}}_{n}(t_{m})={\hat{k}}_{n}(t_{m})-{\bar{\hat{k}}}_{n}$$

where $t_{m}$ denotes the central time instant of the mth azimuth sub-aperture, ${\hat{k}}_{n}\left(t_{m}\right)$ denotes the estimated DR of the nth range bin in the mth azimuth sub-aperture, ${\bar{\hat{k}}}_{n}$ denotes the mean value of the estimated DRs in nth range bin.

From Equations (4) and (5), we obtain

(6) $$\Delta {\hat{k}}_{n}(t_{m})=-\frac{2}{\lambda }a_{y}(t_{m})\sin\beta_{n}-\frac{2}{\lambda }a_{z}(t_{m})\cos\beta_{n},\;1\leq n\leq N$$

where $\beta_{n}$ denotes the target look angle in nth range bin.

The Equation (6) can be rewritten as:

(7) $$\hat{H}\hat{X}=\hat{D}$$

where $\hat{H}=\left[\begin{array}{l} -\frac{2}{\lambda }\sin\beta_{1},-\frac{2}{\lambda }\sin\beta_{1}\\ \vdots \\ -\frac{2}{\lambda }\sin\beta_{N},-\frac{2}{\lambda }\sin\beta_{N} \end{array}\right]$, $\hat{X}={\left[a_{y},a_{z}\right]}^{T}$, $\hat{D}={\left[\Delta {\hat{k}}_{1},\Delta {\hat{k}}_{2},\cdots ,\Delta {\hat{k}}_{N}\right]}^{T}$. The LSE of $\hat{X}$ is:

(8) $$\hat{X}={\left({\hat{H}}^{T}\hat{H}\right)}^{-1}{\hat{H}}^{T}\hat{D}$$

where ${\left[\;•\;\right]}^{T}$ and ${\left[\;•\;\right]}^{-1}$ denote matrix transpose and inverse, respectively.

After ${\left[a_{y},a_{z}\right]}^{T}$ is calculated, the radial accelerations of each sub-aperture in LOS direction can be expressed as:

(9) $$a_{R}(t_{m})=a_{y}(t_{m})\sin\beta +a_{z}(t_{m})\cos\beta$$

Finally, the displacement in LOS direction was acquired by a double integral operation

(10) $$\Delta r=\iint_{t_{m}}a_{R}(t_{m})dt_{m}$$

Note that the unknown constant in the double integral in Equation (10) would result in azimuth position shift for the resulted SAR imagery. To mitigate this problem, the starting time instant for integration should satisfy that $a_{R}\left(t_{m}\right)$ is equal to zero, and then set the selected time instant to be the reference point of a new slow time.

## 3. Proposed IRDM Approach for Sliding Spotlight SAR

In this section, we first state the problems when commonly used stripmap RDM approach is directly applied on sliding spotlight SAR data. Then, the proposed approach is presented.

### 3.1. Problems Statement

Different from stripmap mode, in the spotlight and sliding spotlight mode the azimuth beam is steered during the whole acquisition interval, which results in a time-varying squint angle. Two issues must be taken into consideration when conventional stripmap RD-RIMEE approach is used for sliding spotlight mode SAR data processing. The first issue lies in the sub-aperture DRE estimation step. The segmented sub-apertures corresponding to different azimuth time periods have different theoretical DRs, so the time-varying DRs should be subtracted from the estimated DRs when calculating sub-aperture DRE. The second issue lies in the accurate estimation of time-varying radial accelerations with the obtained range-varying DRE. Both the instability of the airborne platform and the rugged topography can add difficulties in the estimation process; therefore, additional techniques are needed to ensure the accuracy.

### 3.2. IRDM Approach

**Figure 2:**
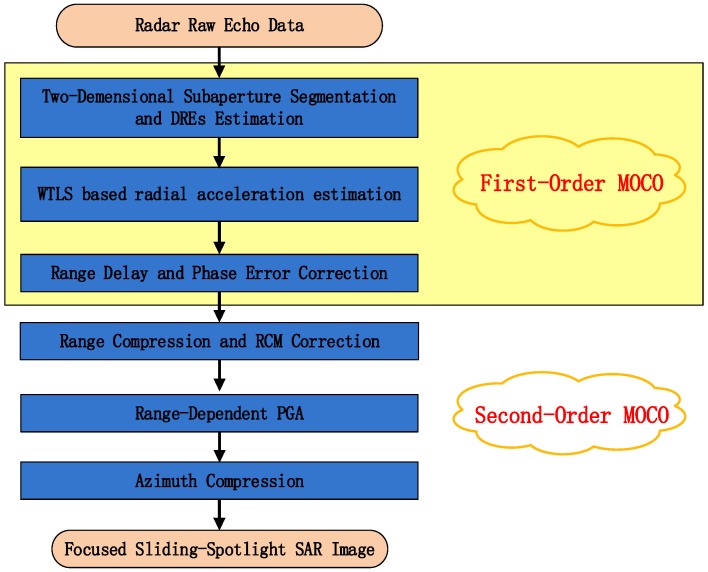
Main flowchart of proposed approach for sliding spotlight Synthetic Aperture Radar (SAR) image formation.

To deal with above mentioned issues, IRDM approach is proposed, the main flowchart of the algorithm for the processing of sliding spotlight data is shown in Figure 2. Compared to the original RDM approach [18], some steps are modified with innovation, which is described in detail as follows:

***—Two dimensional (2D) sub-aperture segmentation and DREs estimation.*** In this step, radar raw data are segmented into overlapped sub-apertures in azimuth, the length of which should be far less than the synthetic aperture length. Second, each azimuth sub-aperture is further separated into multiple overlapped range blocks in range direction. Third, under the assumption that variation of Doppler parameters in each sub-aperture can be ignored, the sub-aperture DRs are estimated via classical MD technique [13]. Due to the azimuth beam steering, the theoretical values of DRs are different along time. Therefore, the sub-aperture DREs cannot be directly obtained as the same with stripmap mode. To solve this problem, in our implementation, the DREs in sliding spotlight mode were calculated as follows:

(11) $$\begin{array}{ll} \Delta {\hat{k}}_{l}^{slide}(t_{m}) & ={\hat{k}}_{l}^{slide}(t_{m})-{\tilde{k}}_{l}^{slide}(t_{m})\\ & ={\hat{k}}_{l}^{slide}(t_{m})-\frac{2\cdot v_{r}^{2}}{\lambda R_{l}}\cos^{2}(\theta_{0}+\theta_{m}) \end{array}$$

where ${\hat{k}}_{l}^{slide}(t_{m})$ denotes the estimated sub-aperture DRE of the lth range block in mth azimuth sub-aperture from the SAR raw data (the DRE of the real trajectory), ${\tilde{k}}_{l}^{slide}(t_{m})$ denotes the corresponding theoretical sub-aperture DRE derived from the nominal trajectory, $R_{l}$ is the referred slant range of the lth range block, $\theta_{0}$ is the central squint angle of the whole acquisition interval, $\theta_{m}$ is the instantaneous beam steering angle in mth azimuth sub-aperture.

***—WTLS based radial acceleration estimation.*** After obtaining the DRE of the 2D sub-apertures, theoretically, $a_{y}(t_{m})$ and $a_{z}(t_{m})$ can be estimated according to Equation (6) using the LSE method. However, in practice, the airborne platform generally undergoes different levels of roll motion during the data acquisition. The amplitude and period of the roll motion is related to the type of the aircraft as well as the wind speed and direction. We also assume a flat ground. Either the unwanted roll motion or the rugged topography can result in errors when calculating the target look angle for different range blocks. This influences the estimation accuracy of $a_{y}(t_{m})$ and $a_{z}(t_{m})$. To solve the above-mentioned problems, drawing on the successful experience of the predecessors [20], a novel WTLS method was chosen to improve the estimation accuracy. Thus, according to Equation (6) and the WTLS method presented in [20], we obtain

(12) $${\hat{X}}_{WTLS}={\left(H^{T}P_{sw}H\right)}^{-1}H^{T}P_{sw}D$$

where ${\hat{X}}_{WTLS}={\left[a_{y}(t_{m}),a_{z}(t_{m})\right]}^{T}$ is the acceleration to be calculated in time $t_{m}$, $H=\left[\begin{array}{l} -\frac{2}{\lambda }\cos\left(\theta (t_{m})\right)\sin\beta_{1},-\frac{2}{\lambda }\cos\left(\theta (t_{m})\right)\sin\beta_{1}\\ \vdots \\ -\frac{2}{\lambda }\cos\left(\theta (t_{m})\right)\sin\beta_{N},-\frac{2}{\lambda }\cos\left(\theta (t_{m})\right)\sin\beta_{N} \end{array}\right]$ is the geometry matrix, $\beta_{n}(n=1,\cdots ,N)$ is the theoretical target look angle corresponding to the scene center of the *n^th^* range block, $D={\left[\Delta {\hat{k}}_{a}(R_{1},t_{m}),\Delta {\hat{k}}_{a}(R_{2},t_{m}),\cdots ,\Delta {\hat{k}}_{a}(R_{N},t_{m})\right]}^{T}$ is the estimated DRE in *N* different range blocks in time $t_{m}$, $P_{sw}=\left(U_{S}U_{S}^{T}\right)W_{T}\left(U_{S}U_{S}^{T}\right)$, $U_{S}$ is constructed from the left singular vectors that correspond to the big singular value of matrix [H D], $W_{T}=\mathrm{diag}\left[w_{1},w_{2},\cdots ,w_{N}\right]$ is the weighting matrix, the weights are determined by the energy of the different sub-apertures.

After obtaining $a_{y}(t_{m})$ and $a_{z}(t_{m})$, the radial accelerations in the LOS direction can be calculated as:

(13) $$a_{R}^{Spot}(t_{m})=a_{y}(t_{m})\cos\left(\theta (t_{m})\right)\sin\beta_{0}+a_{z}(t_{m})\cos\left(\theta (t_{m})\right)\cos\beta_{0}$$

The parameter $\beta_{0}$ in Equation (13) is generally set with the value at the scene center, which can give consideration to the whole range swath. Then, the accelerations $a_{R}^{Spot}(t_{m})$ are up-sampled to be equal with the azimuth sampling points, and finally double integrated to obtain LOS displacements Δr using Equation (10).

***—Range Delay and Phase Error Correction.*** Once the LOS displacements Δr are extracted, the aircraft trajectory is corrected to be a straight line by applying both range delay and phase error correction, which is commonly called first-order MOCO [9,10,11,12]. After the correction, the excessive RCM due to trajectory deviations is removed, which is also the foundation of the following second-order MOCO.

It is noted that the excessive RCM may span dozens or even hundreds of range bins in high-resolution sliding spotlight case. Similar to the MD method [13], it is common practice to apply the proposed first-order MOCO in an iterative manner with coarse-to-fine range resolution, which can significantly improve the accuracy of the final displacements estimate. Typically, not more than six iterations are sufficient to yield a satisfactory result.

***—Second-order MOCO.*** After the first-order MOCO, range compression and RCM correction are implemented. Although the first-order MOCO has removed the excessive RCM completely, it just compensated the phase error at the scene center with limited accuracy. Therefore, a second-order MOCO is needed to compensate the range-dependent residual phase errors. In this paper, a range-dependent PGA is selected [22], which has been tested with a large amount of SAR data.

Once the range-dependent residual phase errors are obtained, the following phase error correction and azimuth compression steps can be easily completed, separately. At last, well focused high-resolution wide-swath sliding spotlight SAR imagery can be obtained.

It is to be noted that the standard spotlight mode can be seen as a special case of the sliding spotlight mode, where the radar beam is also steered. Except for the difference that the beam always illuminates the same region in the spotlight mode, making the synthetic aperture length larger than the sliding spotlight mode. Fortunately, the proposed approach estimates the motion errors in a sub-aperture manner. Within each sub-aperture, the DRE estimation method is universal for spotlight and sliding spotlight mode. So, the proposed approach can also be applied for standard spotlight mode.

## 4. Results and Analysis

To demonstrate the performance of the proposed approach, sliding spotlight mode raw echo data collected by an experimental airborne radar, which was developed by the Institute of Electronics, Chinese Academy of Sciences, is processed. The airborne platform used for the experimental data collection is a small Cessna Citation plane and only equipped with an old built-in INS, whose updated frequency can just provide rough speed and altitude of the plane but cannot be exploited for MOCO. The main system and processing parameters are given in Table 1. Note that the sliding spotlight mode in the experimental system is originally designed with broadside geometry, the small squint angle presented in Table 1 is mainly caused by the yaw motion of the airborne platform. Since the PRF value is greater than the total Doppler bandwidth, ECS algorithm [11], which can integrate with the two-step MOCO approach in the presence of small squint angle, is used to process the sliding spotlight mode raw echo data without azimuth pre-processing step.

### 4.1. Example 1: Land Surface Scene

**Figure 3:**
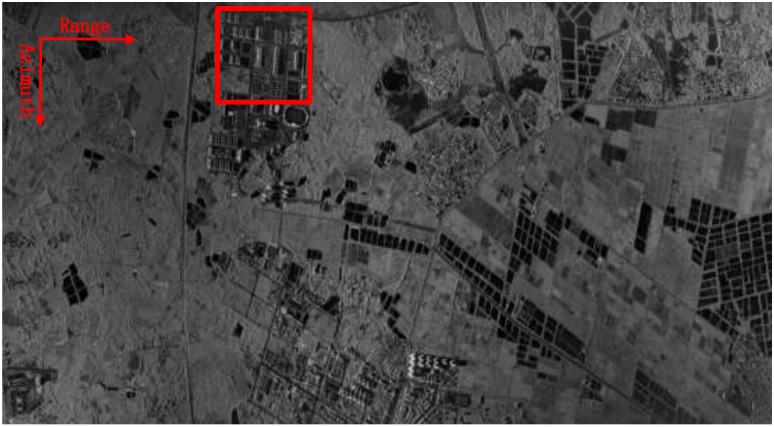
First processed sliding spotlight SAR image via the proposed approach.

The first sliding spotlight SAR image processed by the proposed approach is shown in Figure 3. The original image has a size of 10,240 × 5550 pixels, from which we can see that the entire image is well focused. The theoretical single-look azimuth resolution is about 0.15 m corresponding to the scene center. After multi-look processing, the pixel spacing is about 0.56 m × 0.54 m in the slant-range and azimuth, respectively. In the implementation of the proposed approach, the segmented 2D sub-apertures are half-overlapped and the lengths are set to 512 samples and 2048 samples in range and azimuth. Four iterations are used. After that, the residual phase errors are compensated by second-order MOCO step via range-varying PGA [22].

**Figure 4:**
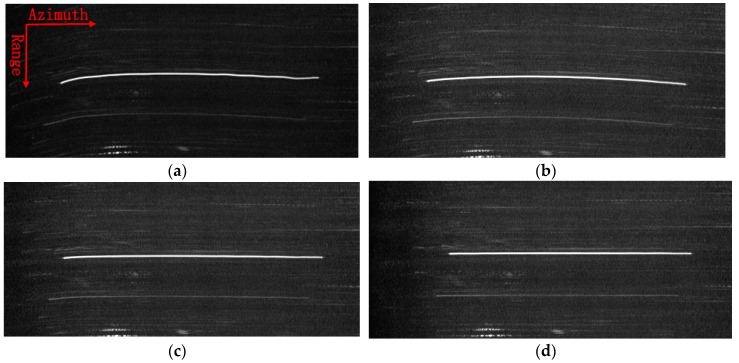
Target trajectory after Range Cell Migration (RCM) correction. (**a**) Without Reflectivity Displacement Method (RDM) approach; (**b**) RDM approach for stripmap mode; (**c**) RDM approach for slide spotlight mode without Weighted Total Least Square (WTLS); (**d**) RDM approach for slide spotlight mode with WTLS.

To show the advantages of the proposed approach, the profile of a dominant target in range compressed and azimuth time domain is displayed in Figure 4 at a size of 220 × 500 pixels. To clearly show the target trajectory, 100 times down-sampling has been done in azimuth. It can be seen that the trajectory without RDM approach shown in Figure 4a is severely curved, due to the unwanted LOS displacements. Furthermore, the curved trajectory is still clearly seen after using stripmap RDM approach. By using the sliding spotlight RDM approach, as shown in Figure 4c, the curved trajectory of the target is nearly corrected. However, a little curve remains. Finally, after integrating WTLS technique in sliding spotlight RDM approach, the curved trajectory is corrected to be a straight line, which is shown in Figure 4d indicating the effectiveness of the proposed approach.

**Figure 5:**
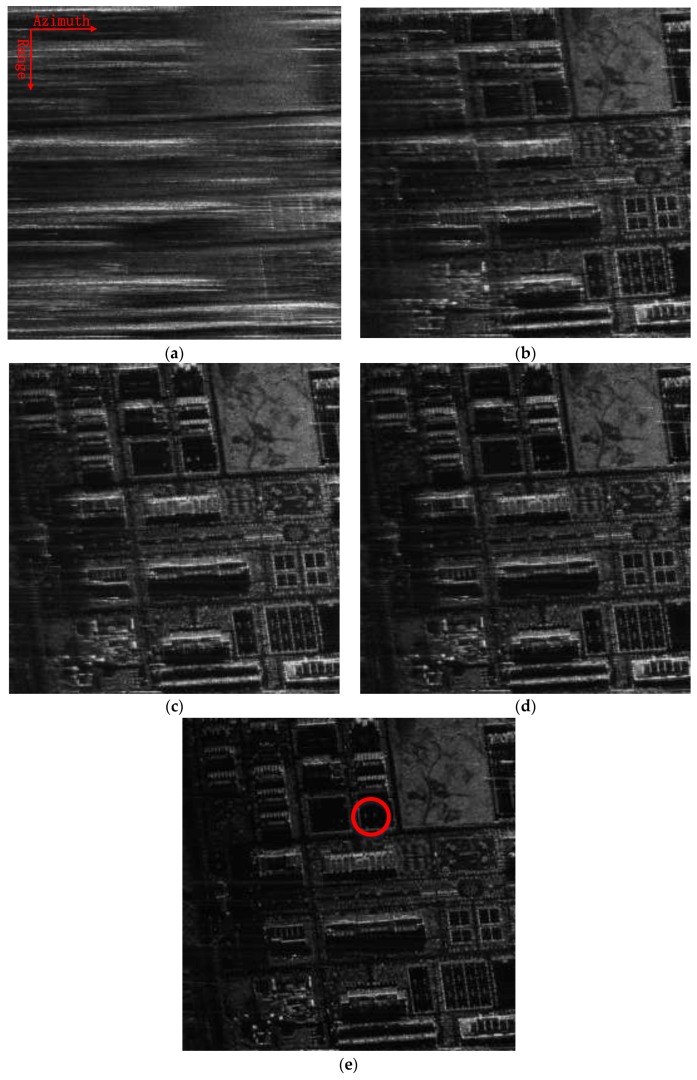
A typical area in Figure 3 are zoomed and compared with the other methods. (**a**) Focused without Motion Compensation (MOCO); (**b**) Focused with only range-varying Phase Gradient Autofocus (PGA); (**c**) Focused combining stripmap RDM and range-varying PGA; (**d**) Focused combining sliding spotlight RDM without WTLS and range-varying PGA; (**e**) Focused combining sliding spotlight RDM with WTLS and range-varying PGA.

The local scenes at a size of 500 × 500 pixels, marked with red squares in Figure 3, are zoomed into and compared with other methods, which are shown in Figure 5. Both the images processed without MOCO and with only second-order MOCO are severely defocused. The processing result by combining the stripmap RDM approach and range-varying PGA is shown in Figure 5c, which has a much higher quality than Figure 5a,b. However, the resulting image is still blurred and needs to be improved. Although the quality of image shown in Figure 5d is improved by combining the sliding spotlight RDM approach and range-varying PGA, the defocusing phenomena still exists, especially in the left side of the image, where the flight is bumpy and the trajectory deviations are larger. Finally, the image is well focused in Figure 5e, where the roof of the buildings can be clearly distinguished. Meanwhile, the low backscattered areas have higher contrast in Figure 5e, clearly showing the advantages of the proposed algorithm. In addition, image contrast (IC) value and image entropy (IE) value, which generally represent good indicators of the general image focusing, are used to gauge the success of the proposed approaches [15,16]. As reported in Table 2, the image presented in Figure 5e shows the largest IC and the smallest IE value, which is in accordance to our image visual analysis.

**Figure 6:**
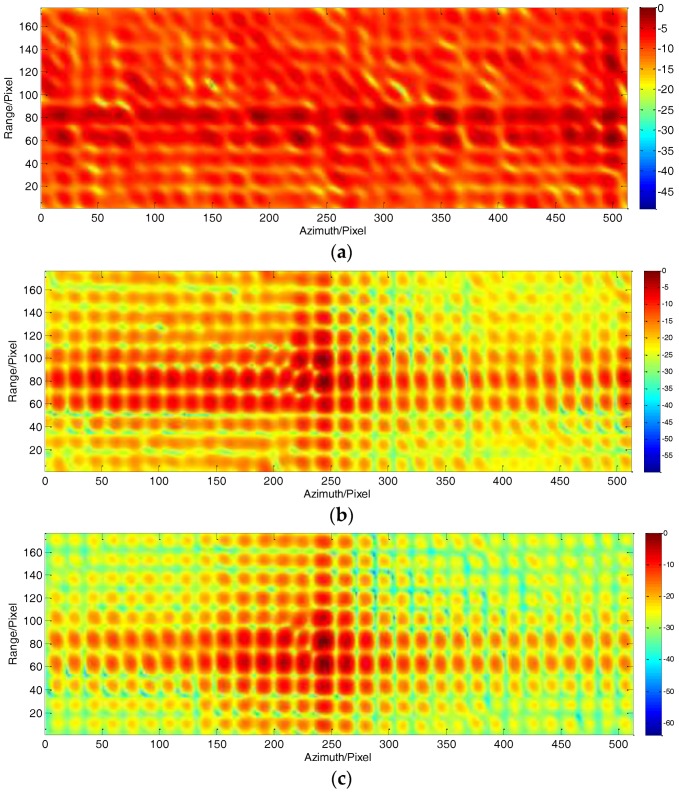

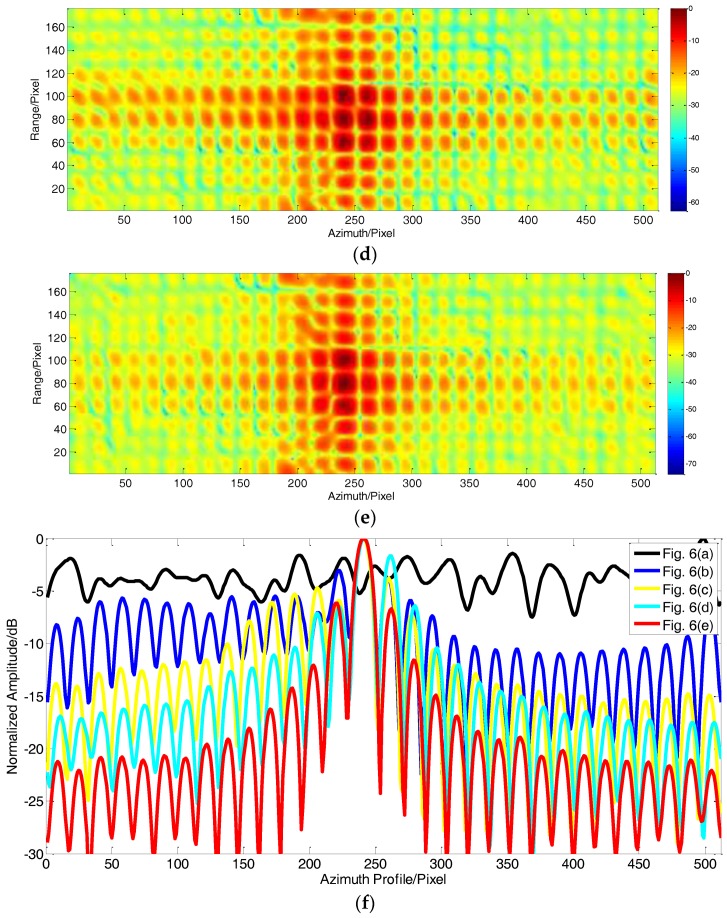
Point spread functions and azimuth profiles of bight targets in Figure 5. (**a**) Focused without MOCO; (**b**) Focused with only range-varying PGA; (**c**) Focused combining stripmap RDM and range-varying PGA; (**d**) Focused combining sliding spotlight RDM without WTLS and range-varying PGA; (**e**) Focused combining sliding spotlight RDM with WTLS and range-varying PGA; (**f**) The azimuth profiles of the brightest target in Figure 6a–e.

To make a precise study of the performance enhancement gained using the proposed algorithm. A small local area with bright targets, which is signed with red circle in Figure 5e, is chosen for quantitative analysis. The two-dimensional point spread function of the bright targets with different methods are presented in Figure 6a–e. From these figures, we can see that the proposed method gets the best performance, which has the highest energy concentration and thus has the widest dynamic range. Furthermore, the azimuth profiles of the brightest target in Figure 6a–e are plotted together and given in Figure 6f. From the figure, we can see that target cannot be distinguished in Figure 6a. After some MOCO operations, the target can be found by searching the peak value in Figure 6b–e. However, the side-lobes in Figure 6b–d are relatively high and asymmetric, which can easily cause the false alarm targets. Finally, the azimuth profile in Figure 6e has the regular shape, which is highly asymmetric and decreased monotonously. Although the peak to side-lobe ratio is still higher than the standard value, the phenomenon can be caused by the non-ideal point target and system noise.

### 4.2. Example 2: Coastal Zone

**Figure 7:**
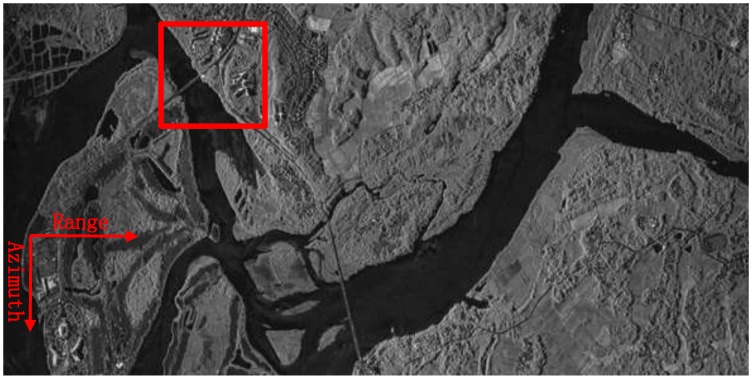
Second processed sliding spotlight SAR image via the proposed approach.

To give stronger demonstration of the proposed approach, another sliding spotlight SAR dataset acquired with the same system was processed. In this example, a typical coastal zone located at Hainan province was chosen to be the SAR illuminated area. The processed image by the proposed approach is shown in Figure 7. The original image has a size of 10,000 × 5000 pixels, from which we can see that the entire image is also well focused. The operational parameters used during SAR data processing is the same as the first example.

**Figure 8:**
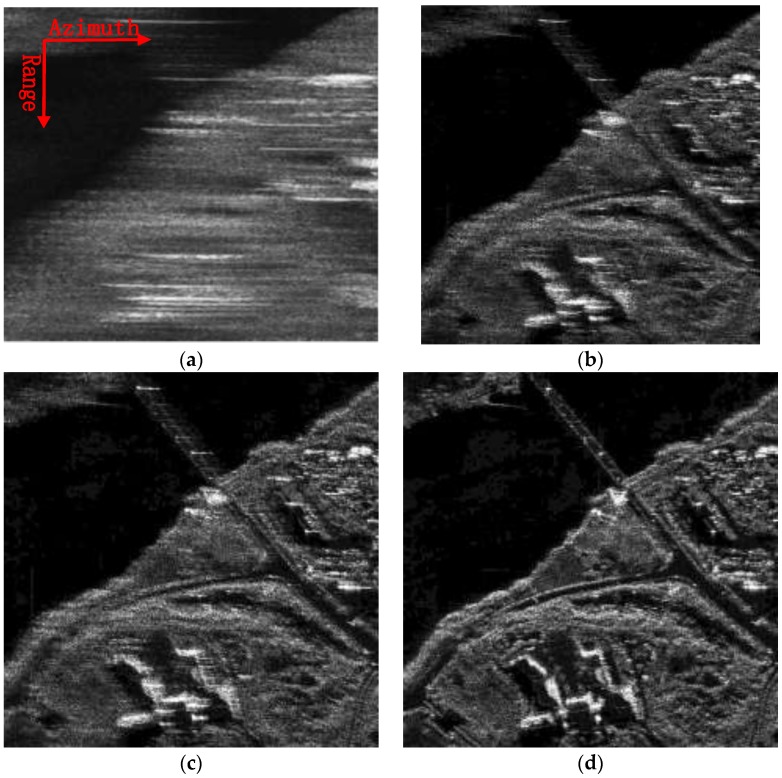

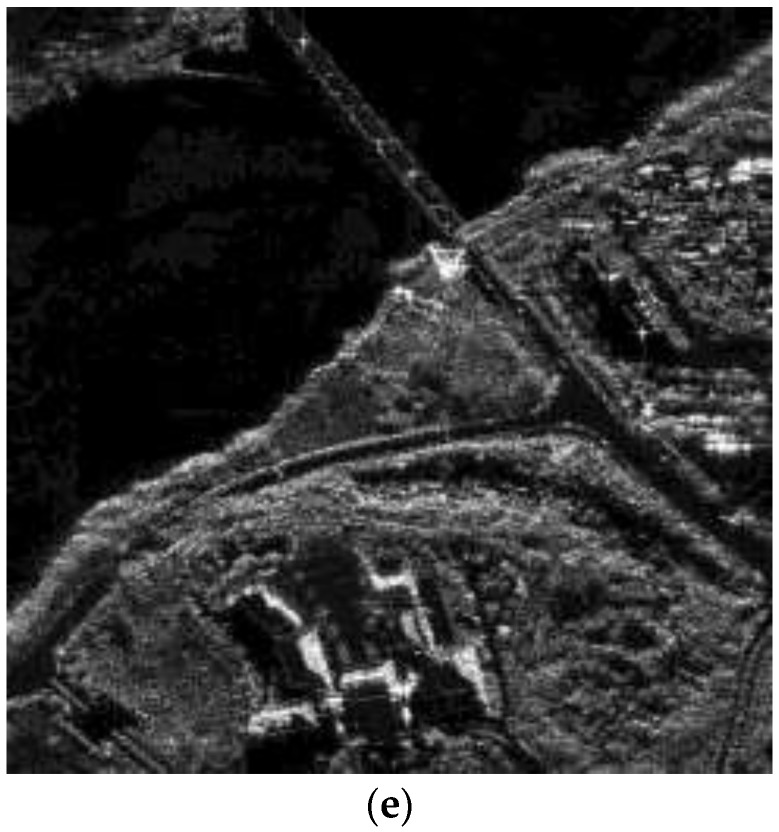
A typical area in Figure 6 are zoomed and compared with the other methods. (**a**) Focused without MOCO; (**b**) Focused with only range-varying PGA; (**c**) Focused combining stripmap RDM and range-varying PGA; (**d**) Focused combining sliding spotlight RDM without WTLS and range-varying PGA; (**e**) Focused combining sliding spotlight RDM with WTLS and range-varying PGA.

The local scenes at a size of 500 × 500 pixels, composed of water, bridge and land, marked with red squares in Figure 7, are zoomed into and compared with other methods, which are again shown in Figure 8. As the same with the first example, both the images processed without MOCO and with only second-order MOCO are severely defocused. As shown in Figure 8a,b, an interpretation of the objects in the image will be failed. After applied the different RDM approach, as shown in Figure 8c–e, the image qualities are gradually improved. Among them, Figure 8e, processed by the proposed approach, achieves the best imaging results, and thus validates the superiority of the proposal.

In addition, the IC and IE value are also calculated and reported in Table 3, the image presented in Figure 8e shows the largest IC and the smallest IE value, which sustains the forward analysis.

## 5. Conclusions

In this article, a novel raw data-based MOCO approach is presented for sliding spotlight mode, which can accurately estimate the displacements in LOS direction caused by motion errors. Together with a traditional second-order MOCO step, well focused high-resolution sliding spotlight SAR imagery can be obtained. To demonstrate the performance of the proposed approach, two airborne sliding spotlight mode real datasets with high azimuth resolution are processed. The imaging results show that the proposed approach is effective and significantly improves the image quality, which has great potential for high-resolution sliding spotlight SAR data processing with low-accuracy navigation equipment.

## Figures and Tables

**Table 1:** Main system and processing parameters for the experiment.

Parameters	Value	Parameters	Value
Carrier frequency	5.4 GHz	Central look angle	54°
System PRF	2000 Hz	Average speed	135 m/s
Antenna length	0.624 m	Acquisition interval	42.48 s
Pulse Bandwidth	200 MHz	Maximum steering angle	±6.4°
Sampling frequency	266.7 MHz	Squint angle	−1.2°

**Table 2:** Image Contrast (IC) and Image Entropy (IE) of the typical area in Figure 5.

Method	IC	IE
Without MOCO	0.6435	13.5625
Range-varying PGA	0.8111	13.3077
Stripmap RDM + Range-varying PGA	0.8453	13.2287
Sliding spotlight RDM without WTLS + Range-varying PGA	0.8504	13.1893
Sliding spotlight RDM with WTLS + Range-varying PGA	0.8647	13.1597

**Table 3:** IC and IE of the typical area in Figure 8.

Method	IC	IE
Without MOCO	0.6664	13.8767
Range-varying PGA	0.8447	13.3145
Stripmap RDM + Range-varying PGA	0.8619	13.2037
Sliding spotlight RDM without WTLS + Range-varying PGA	0.8982	13.1880
Sliding spotlight RDM with WTLS + Range-varying PGA	0.9191	13.0849
